# Pulmonary Rehabilitation Reduces Subjective Fatigue in COPD: A Responder Analysis

**DOI:** 10.3390/jcm8081264

**Published:** 2019-08-20

**Authors:** Maarten Van Herck, Jeanine Antons, Jan H. Vercoulen, Yvonne M. J. Goërtz, Zjala Ebadi, Chris Burtin, Daisy J. A. Janssen, Melissa S. Y. Thong, Jacqueline Otker, Arnold Coors, Mirjam A. G. Sprangers, Jean W. M. Muris, Judith B. Prins, Martijn A. Spruit, Jeannette B. Peters

**Affiliations:** 1 REVAL Rehabilitation Research Center, BIOMED Biomedical Research Institute, Faculty of Rehabilitation Sciences, Hasselt University, 3590 Diepenbeek, Belgium; 2 Department of Research and Development, CIRO+, Center of Expertise for Chronic Organ Failure, 6085 NM Horn, The Netherlands; 3 Radboud University Medical Center, Radboud Institute for Health Sciences, Department of Pulmonary Diseases, 6525 GA Nijmegen, The Netherlands; 4 Radboud University Medical Center, Radboud Institute for Health Sciences, Department of Medical Psychology, 6525 GA Nijmegen, The Netherlands; 5 Centre of Expertise for Palliative Care, Maastricht University Medical Center, 6229 HX Maastricht, The Netherlands; 6 Department of Medical Psychology, Amsterdam University Medical Centers, Location AMC, 1105 AZ Amsterdam, The Netherlands; 7 Member of Lung Foundation Netherlands, 3818 LE Amersfoort, The Netherlands; 8 Member of Patient Advisory Board, Radboud University Medical Center, 6525 GA Nijmegen, The Netherlands; 9 Department of General Practice, CAPHRI Research Institute, 6229 HX Maastricht, The Netherlands; 10 Department of Respiratory Medicine, Maastricht University Medical Center, 6229 HX Maastricht, The Netherlands; 11 NUTRIM School of Nutrition and Translational Research in Metabolism, 6229 ER Maastricht, The Netherlands

**Keywords:** pulmonary rehabilitation, COPD, fatigue, quality of life, responder analysis

## Abstract

To date, it remains unknown which patients report a clinically-relevant improvement in fatigue following pulmonary rehabilitation (PR). The purpose of this study was to identify and characterize these responders. Demographics, lung function, anxiety (anxiety subscale of the 90-item symptom checklist (SCL-90-A)), depression (Beck depression inventory for primary care (BDI-PC)), exercise tolerance (six-minute walking distance test (6MWD)), and health status (Nijmegen clinical screening instrument (NCSI)) were assessed before and after a 12-week PR programme. Fatigue was assessed using the checklist individual strength (CIS)-Fatigue. Patients with a decline ≥ 10 points (minimally clinically important difference, MCID) on the CIS-Fatigue were defined as responders. Chronic obstructive pulmonary disease (COPD) patients (*n* = 446, 61 ± 9 years, 53% male, forced expiratory volume in 1 s (FEV1) 43% ± 18% predicted, 75% severe fatigue) were included. Mean change in fatigue after PR was 10 ± 12 points (*p* < 0.01) and exceeded the MCID. In total, 56% were identified as fatigue responders. Baseline CIS-Fatigue (45 ± 7 vs. 38 ± 9 points, respectively, *p* < 0.001) and health-related quality-of-life (HRQoL; *p* < 0.001) were different between responders and non-responders. No differences were found in demographics, baseline anxiety, depression, lung function, 6MWD, and dyspnoea (*p*-values > 0.01). Responders on fatigue reported a greater improvement in anxiety, depression, 6MWD, dyspnoea (all *p*-values < 0.001), and most health status parameters. PR reduces fatigue in COPD. Responders on fatigue have worse fatigue and HRQoL scores at baseline, and are also likely to be responders on other outcomes of PR.

## 1. Introduction

Chronic obstructive pulmonary disease (COPD) is a common preventable and treatable disease, which is characterized by persistent airflow limitation [1]. Most COPD-related studies only focus on respiratory-related symptoms (i.e., dyspnoea, and to a lesser extent also phlegm, wheezing, cough, and/or chest tightness) [2]. Nevertheless, more than half of the patients with COPD suffer from severe fatigue [3]. This extra-pulmonary symptom is defined as “the subjective feeling of tiredness, exhaustion or lack of energy, that occurs on a daily basis” [4]. Severe fatigue may have a substantial impact on functional impairment, physical activity, and quality of life (QoL) in patients with COPD [3,5,6,7], and is often described as invalidating, and results in care dependency [8]. Furthermore, fatigue is also related with mortality [9], morbidity, exacerbation-related hospitalization, and length of hospital stay [10,11]. Therefore, reducing fatigue has been put forward by patients as one of the priorities for respiratory research [12]. A four-year observational study on fatigue in patients with COPD reported that severe fatigue doubled in patients with mild to severe COPD despite optimal COPD care [13]. Hence, patients need more than just standard COPD treatment to improve fatigue. Nowadays more complex patients with a high symptom burden and multiple limitations (during daily living) are referred to a multidisciplinary pulmonary rehabilitation (PR) programme [14], which is defined as “a comprehensive intervention based on a thorough patient assessment followed by patient-tailored therapies which include, but are not limited to, exercise training, education, and behaviour change, designed to improve the physical and psychological condition of people with chronic respiratory disease and to promote the long-term adherence of health-enhancing behaviours” [15]. PR has shown positive effects on daily symptoms of dyspnoea, exercise tolerance, and QoL in patients with COPD [16]. Houben-Wilke and colleagues recently showed that the item ‘energy’ of the COPD assessment test (CAT) improves with the greatest effect size after PR [17]. A single CAT item, however, is too limited to truly assess the impact of PR on fatigue. A common multi-dimensional scale to evaluate fatigue is the subjective fatigue subscale of the checklist individual strength (CIS-Fatigue) [18]. Peters and colleagues already reported a significant mean improvement in CIS-Fatigue score following 12-week of PR in patients with COPD [19]. However, it remains unknown which patients show a clinically relevant improvement in fatigue. The aims of this study were (1) to determine the proportion and characteristics of COPD patients with a clinically relevant improvement in fatigue scores following PR; (2) to assess the impact of PR in responders and non-responders on fatigue; and (3) its relation with change in possible contributing factors to fatigue.

## 2. Methods

### 2.1. Study Design and Participants

A secondary analysis of data, prospectively collected at the start and the end of a pulmonary rehabilitation (PR) programme at the University Lung Centre Dekkerswald of the Radboud University Medical Center (Radboudumc; Nijmegen, The Netherlands), was conducted [19]. Chest physicians referred these outpatients to PR because of a persistent high symptom burden, problems in activities of daily living, difficulties to cope with/adapt to their disease or a combination of these, despite optimal COPD care. Eligibility criteria were (1) diagnosis of COPD based on the Global Initiative for Chronic Obstructive Lung Disease (GOLD) with a post-bronchodilator forced expiratory volume in 1 s (FEV_1_) to forced vital capacity (FVC) ratio < 0.7 [1], (2) completion of the 12-week PR programme, (3) pre-PR data available regarding gender, age, weight, and lung function (to determine diagnosis of COPD and reference values), and (4) pre- and post-PR data available regarding subjective fatigue (checklist individual strength-fatigue; CIS-Fatigue). The Medical Ethical Committee of the Radboudumc approved this retrospective study of data collected during usual care (reference: 2018-4994).

### 2.2. Pulmonary Rehabilitation

Patients underwent a 12-week (five day/week) inpatient multidisciplinary PR programme as part of usual care. Eight disciplines (pulmonologist, psychologist, physiotherapist, nurse, dietician, psychomotor therapist, social worker, and enhanced art therapist) were involved in the PR programme. Based upon comprehensive health status assessments and clinical interviews by seven disciplines (the art therapist did not participate in the PR assessment), individual goals were set for the PR programme. This multidisciplinary and individualized treatment programme consisted of a training programme, education sessions, group therapy, and individual therapy, as described in the American Thoracic Society (ATS) and European Respiratory Society (ERS) statement regarding PR [15]. Every three weeks, the treatment progress was evaluated by representatives of the eight disciplines and with the patient. If needed, the treatment programme was adapted.

### 2.3. Assessments and Questionnaires

Demographical, clinical, and health status features were assessed during the comprehensive baseline assessment. These assessments and clinical interviews were distributed over two consecutive days, and took place four weeks prior to the start of the PR programme on average.

#### 2.3.1. Demographical Features

The following demographical data were systematically registered at baseline: age, gender, weight, height, tobacco use (non-/ex-/smoker), time since COPD diagnosis (≤ 10/> 10 years), self-reported comorbidities, and education level. The latter was classified in three groups: high, middle, and low based upon the Dutch classification system according to Verhage [20]. 

#### 2.3.2. Clinical Features

Body composition was measured as fat free mass index (FFMi; kg/m^2^) by bio-electrical impedance analysis (BIA) and body mass index (BMI; kg/m^2^). BMI was calculated and classified in four subgroups: underweight (BMI < 21) [21], normal weight (21 ≤ BMI < 25), overweight (25 ≤ BMI < 30), and obese (BMI ≥ 30) [22]. Lung function was evaluated with post-bronchodilator spirometry according to the ATS/ERS guidelines. Based upon the degree of airflow limitation, patients were classified into four subgroups (GOLD stages): GOLD grade I (FEV_1_ ≥ 80% predicted), GOLD grade II (50 ≤ FEV_1_ < 80% predicted), GOLD grade III (30 ≤ FEV_1_ < 50% predicted), and GOLD grade IV (FEV_1_ < 30% predicted) [1]. Exercise tolerance was measured using a six-minute walking distance test (6MWD) according to ERS/ATS technical standards [23]. The reference values for healthy elderly subjects established by Troosters and colleagues were used to express the distance as a percentage of the predicted value [24]. A score of < 70% predicted was used to classify patients as having an ‘impaired exercise tolerance’ [25]. An improvement of 30 m or more was defined as the minimally clinically important difference (MCID) [23,26]. The quadriceps muscle strength of both legs (maximal isometric muscle force of the knee extensors) was measured using a handheld dynamometer (MicroFET2, Biometrics, Almere, The Netherlands). The highest value was reported. The anxiety subscale of the 90-item symptom checklist (SCL-90-A) was used to measure generalized anxiety in addition to dyspnoea-specific anxiety, which is measured with the Nijmegen clinical screening instrument (NCSI; sub-domain dyspnoea emotions). The SCL-90-A is a self-reported questionnaire and consists of 10 questions on a five-point scale ranging from 1 (‘not at all’) to 5 points (‘extremely’) [27,28]. The SCL-90-A scores range from 10 to 50 points, and a higher score indicates more clinical symptoms of anxiety. A score of ≥ 23 points was used to classify patients as having a ‘level of anxiety more than average’ [29,30].

#### 2.3.3. Health Status and Clinical Features Obtained via NCSI

The health status of patients was assessed using the NCSI. The NCSI is an empirically-composed battery of existing instruments (including the CIS-Fatigue, Beck depression index for primary care, dyspnoea visual analogue scale, sickness impact scale, and others), such that health status is measured in detail by a minimum of items [31]. The NCSI measures 24 aspects of health status that for research purposes are aggregated into the following eight subdomains: subjective dyspnoea, dyspnoea emotions, subjective fatigue, subjective impairment, behavioural impairment, general quality of life (QoL), health-related quality of life (HRQoL), and satisfaction with relations. Table 1 shows the instruments measuring each sub-domain. A higher score on a subdomain of the NCSI indicates a worse health status on the corresponding subdomain [32].

Subjective fatigue was measured by the subjective fatigue subscale of the CIS-Fatigue [18]. The CIS-Fatigue is a standardized and validated questionnaire that has been used in healthy subjects [38] and among various patient populations [39,40], such as COPD [3,31]. The CIS-Fatigue consists of eight items scored on a seven-point Likert scale. Scores range from 8 to 56 points. A score of ≤ 26 points indicates normal fatigue, scores between 27 and 35 points indicate mild fatigue, and a score of ≥ 36 points indicates severe fatigue [18,41]. The MCID of the CIS-Fatigue in patients with COPD is 10 points [13]. Based on the MCID of the CIS-Fatigue, responders and non-responders on fatigue were classified. Patients with a decline ≥ 10 points on the CIS-Fatigue following PR were defined as responders. Patients with a decline < 10 points were defined as non-responders. 

The global dyspnoea burden of patients over the past four weeks was scored using the dyspnoea visual analogue scale (dyspnoea VAS) at the comprehensive baseline assessment four weeks prior to the start of the PR programme. Patients indicated the shortness of breath they felt most days during the past four weeks. Scores of the dyspnoea VAS range from 0 (‘no breathlessness’) to 10 points (‘worst breathlessness possible’) [31].

Depression was measured using the Beck depression inventory for primary care (BDI-PC). The BDI-PC consists of seven items scored on a four-point Likert scale (0–3). Scores range from 0 to 21 points, and a higher score indicates more clinical symptoms of depression. A score of ≥ 4 points was used to categorize patients as ‘depressed’ and signify that a detailed psychiatric evaluation may be warranted [36,42].

#### 2.3.4. Collection of Post-PR Data

The following post-treatment data were collected in the week after discharge: weight, FFMi, BMI, lung function, exercise tolerance, quadriceps muscle strength, anxiety, and health status (NCSI; including subjective fatigue, dyspnoea and depression). 

### 2.4. Statistical Analysis

Statistical analyses were conducted using SPSS v25.0 (IBM Corp., Armonk, NY, USA). Data were presented as mean ± standard deviation (SD), or frequencies and proportions, where appropriate. A ‘responder analysis’ was conducted to determine differences in baseline characteristics and impact of PR between ‘responders’ and ‘non-responders’ on fatigue. Patients with normal fatigue at baseline where excluded from the ‘responder analysis’. Differences between ‘responders’ and ‘non-responders’ on fatigue upon baseline characteristics and impact on PR (change scores following PR) were analysed by unpaired *t*-test or the non-parametric pendant (Mann–Whitney U test) for continuous data, where appropriate. Categorical data were analysed with the chi-square test or Fisher’s exact test, where appropriate. If significant, a post-hoc comparison of the chi-square test was performed, and significant Bonferonni-adjusted *p*-values were generated as correction for multiple comparison. To examine the effectiveness of the PR, paired *t*-tests (or Wilcoxon signed-rank tests) were used to compare pre- and post-PR data, where appropriate. Bivariate correlations were assessed by Pearson or Spearman’s rank correlation, where appropriate, to assess the relationship between change in fatigue and change in possible contributing factors to fatigue. Cohen’s d was used to interpret the effect size of correlation coefficients. A correlation coefficient of 0.10~<0.30 represents a weak/small association, 0.30~<0.50 is considered a moderate correlation; and ≥0.50 is thought to represent a large/strong correlation [43]. Change scores (Δ) were calculated as post-PR scores minus pre-PR scores. The level of significance was set at 0.01 for all statistical tests (two-tailed).

## 3. Results

### 3.1. Patient Characteristics

Between July 2002 and July 2013, 459 COPD patients completed the inpatient PR, of whom 446 patients were eligible for inclusion. Reasons for exclusion were absence of data regarding lung function and weight at baseline (*n* = 10 and *n* = 3, respectively). Most patients with COPD had a low level of education, were former smokers, had (very) severe COPD, and reported one or more comorbidities. Additionally, 78.4% had a 6MWD < 70% predicted, indicating an impairment in exercise tolerance [25]. At the start of the PR, 93.5% (417 out of 446) reported (abnormal) fatigue, 18.6% mild fatigue, and 74.9% severe fatigue. Clinical indications for anxiety and depression were found in 21.6% and 38.6% of the patients, respectively. Table 2 shows these characteristics in detail.

### 3.2. Effects of PR on Subjective Fatigue

**Figure 1:**
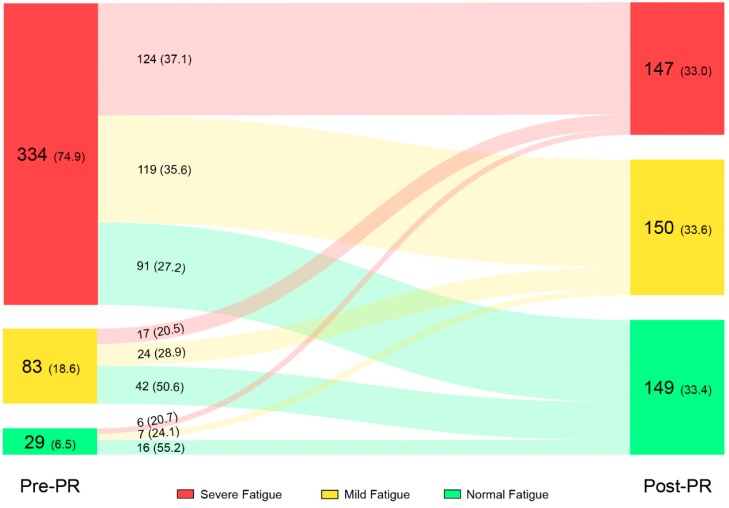
Prevalence of patients with normal, mild and severe fatigue before and after PR, and change in fatigue level following PR. Data in the figure is presented as number of subjects (%). Abbreviations: PR, pulmonary rehabilitation.

Mean CIS-Fatigue score improved significant and clinically relevant compared to baseline (−10.4 ± 11.7 points; *p* < 0.001: Online Table S1). The prevalence of severe fatigue decreased from 74.9% at the start of the PR to 33.0% after the PR programme. In addition, the proportion of patients with normal fatigue increased from 6.5% to 33.4%. From the patients with severe fatigue at baseline, 37.1% (124 out of 334) reported severe fatigue after PR. The proportion of patients with normal, mild, and severe fatigue before and after PR, the flow of patients following PR, and the direction of change can be found in Figure 1.

### 3.3. Effects of PR on Other Outcome Measures

PR significantly improved exercise tolerance (6MWD, +57.6 ± 73.2 m), quadriceps muscle strength (+26.0 ± 64.0 Nm), anxiety (−3.0 ± 5.7 points), and health status measured with the NCSI (all *p*-values < 0.001), including depression (−1.5 ± 2.6 points) and dyspnoea (−1.1 ± 2.1 points). Please see Online Table S1 for all details.

### 3.4. Relationship between Change in Fatigue and Change in Other Outcomes

The change in fatigue was weak to moderate, and significantly correlated with the changes in anxiety (*r* = 0.243, *p* < 0.001), depression (*r* = 0.276, *p* < 0.001), 6MWD (% predicted; *r* = −0.323, *p* < 0.001), dyspnoea (*r* = 0.321, *p* < 0.001), HRQoL (*r* = 0.424, *p* < 0.001), and health status in general (all *p*-values ≤ 0.005). All details can be found in Online Table S2.

### 3.5. Responders versus Non-Responders on Fatigue

**Figure 2:**
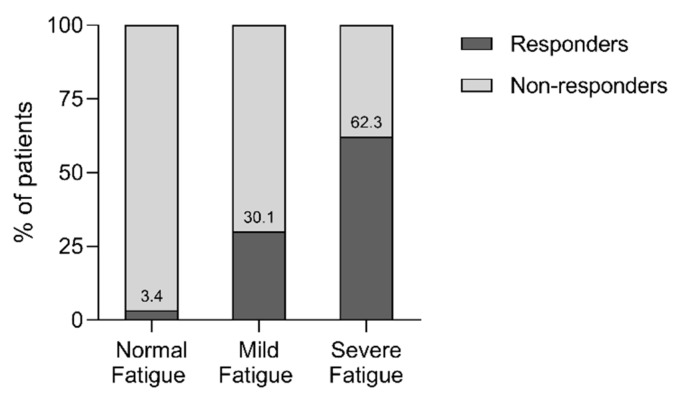
Distribution of responders and non-responders stratified for fatigue level at baseline. A statistically significant association between degree of fatigue at baseline and (non-) responders on fatigue was observed, *χ*^2^ (2, *n* = 446) = 57.445, *V* = 0.359, and *p* < 0.001. Abbreviations: *n*, number of subjects; *V*, Cramer’s *V*.

52.5% of all patient (234 out of 446), and 55.9% of patients with abnormal (mild or severe) fatigue at baseline (233 out of 417) qualified as responders on fatigue after PR. A minority of patients with normal fatigue and mild fatigue at baseline were classified as responders on fatigue (3.4% and 30.1%, respectively), in comparison to 62.3% of the initial severe fatigue group (Figure 2). 

### 3.6. Responder Analysis

Results of the responder analysis (*n* = 417) upon differences between responders and non-responders on fatigue at baseline can be found in Table 3. At baseline, fatigue and HRQoL were worse in responders versus non-responders. Baseline demographics, body composition, lung function, exercise tolerance, quadriceps muscle strength, anxiety, and health status (including dyspnoea and depression) were comparable.

Responders on fatigue showed a significantly greater improvement in anxiety, depression, dyspnoea, exercise tolerance, and the majority of all health status parameters (with the exception of behaviour impairment) compared to non-responders (all *p*-values ≤ 0.01; Table 4).

## 4. Discussion

This is the first study to show that about half of the patients with COPD have a clinically relevant improvement in fatigue following PR, and that the responders on fatigue (patients with ΔCIS-Fatigue ≥ 10 points) also had greater improvements in exercise tolerance, anxiety, and health status (including depression, dyspnoea, and HRQoL) compared to non-responders. 

The prevalence of fatigue in patients with COPD is high at the start of an inpatient PR programme. Approximately three-quarters of all patients referred for PR suffered from severe fatigue at baseline. These findings are in line with findings of previous studies in patients referred for PR [44,45]. Fortunately, an interdisciplinary PR programme reduces fatigue in patients with COPD, as already found by Peters and colleagues [19] and others (all relative small samples) [46,47,48,49,50]. Interestingly, about half of the COPD patients with mild or severe fatigue who completed the 12-week inpatient PR programme (55.9%) showed a clinically relevant improvement in fatigue. This was even higher in patients with severe fatigue (62.3%), and cannot be attributed to regression to the mean only. Indeed, mild or severe fatigue for sure will not improve following usual COPD care. Several studies have already reported that fatigue increases in patients with COPD, despite optimal COPD care [13,19,51,52]. Therefore, these findings emphasize that complaints of mild or severe fatigue may be a reason for referral to PR [53]. In total, 30 patients showed an increase in level of fatigue following PR (Figure 1). This is remarkable and several possible explanations can be given. First of all, this increase can be attributed to regression to the mean, whereas most of these patients scored relatively low at baseline. Other explanations are physical deterioration due to a COPD-related exacerbation during PR or an increase in fatigue as result of an awareness-raising process/change in coping style which occurred during PR [54].

Several weak but significant correlations were found between change in fatigue and change in possible contributing factors to fatigue following PR (such as anxiety, depression, exercise tolerance, dyspnoea, and others). Two other small-scale studies found comparable results for depression, exercise tolerance, and QoL, but not for anxiety [47,55]. Fatigue in COPD is above all the result of complex interactions between physiological, behavioural, systematic, and psychological processes which are different from person to person [56,57]. This clarifies the weak to moderate, but significant correlations found. Based on the currently available data, it is difficult to understand which PR ingredients have contributed to the reduction in fatigue [14]. Moreover, the current study design does also not allow us to derive causality between the changes in fatigue and the changes in anxiety, depression, dyspnoea, exercise tolerance, and health status. Furthermore, findings of our study illustrated that responders on fatigue achieved greater improvement on other outcomes in PR. The role of fatigue (and its improvement) in the rehabilitation process and outcomes of PR needs to be further investigated.

PR seems to be an effective strategy to reduce fatigue in the majority of COPD patients, whereas previous studies showed that the mean fatigue did not decrease after six months and after one year of usual care [19,51]. Indeed, patients with severe fatigue at baseline benefit the most in terms of fatigue (Figure 2). The effectiveness of the PR programme upon fatigue may even be underestimated, as patients with normal fatigue are included in the study because of a regression to the mean/floor effect as there is less room to improve and a limiting ability to detect response on fatigue in these patients [58]. Yet, one-third of patients with severe fatigue at baseline were still classified as non-responders on fatigue, despite their room for improvement (Figure 2). Non-responders on fatigue were also poor responders on the PR programme in general, regardless of comparable exercise tolerance, dyspnoea, clinical symptoms of anxiety and depression, and health status at the start of the inpatient PR programme. Therefore, these patients may require additional (or more intensified) interventions to reduce fatigue (and its possible contributing factors) [56,59]. Given the relevance of fatigue in patients with COPD, it is important to optimize PR to reduce fatigue in these patients. Our results show that identification of potential responders and non-responders on fatigue at baseline is difficult. Besides differences on fatigue and HRQoL, no other differences were found at baseline (Table 3). Early detection of non-responders during PR (by mid-term evaluation of fatigue and its perpetuating factors) may offer opportunities to optimize PR for these patients.

### Strengths, Limitations, and Clinical Implications

This study investigates fatigue in COPD, a patient-initiated and clinically relevant topic, that despite the high prevalence (and relevance) of fatigue has been neglected in research [12,53]. All patients underwent a broad health status assessment (including anxiety and depression) that resulted in an individualised treatment plan followed by an interdisciplinary PR programme. The current findings demonstrate that fatigue is highly prevalent in COPD patients referred for PR, and emphasize the importance of the use of a fatigue questionnaire (such as the CIS-Fatigue). This study is the first that has included a broad range of possible contributing factors to fatigue. Fatigue has a substantial impact on patients with COPD, and is therefore an important target for treatment [3,5,6]. Our results show that an interdisciplinary PR programme reduces fatigue in patients with COPD, in which usual care (optimal COPD care) does not succeed. To date, a holistic and strongly individualised approach seems to be the most appropriate manner to treat fatigue in COPD because of its multifactorial nature [8,15,53]. Not only an impaired exercise tolerance or high symptom burden, but also severe fatigue may be an indication for PR. This secondary, retrospective analysis of a large prospective cohort has also several limitations. First of all, this study design may affect the validity of the results. Second, several systematic, behavioural, and demographic factors, such as data regarding a patient’s exacerbation rate (previous 12 months), physical activity, sleep quality, medication intake, coping style, and motivation were not available in the current study. These variables may have influenced patients’ potential to achieve a MCID on fatigue. Third, it is suspected that less-motivated patients (but also the more severe or unstable patients) were not represented in this analysis, since participation in the PR was voluntary. Fourth, the influence of fatigue on PR completion cannot be determined, whereas only data of PR completers were available. It is suspected that fatigue may have an influence on PR completion [57]. Fifth, data was collected over a period of 11 years (July 2002 to July 2013), and PR has optimized over the years with greater improvements as a consequence. However, no differences in change scores were found between the 2002 and 2013 cohort upon any variable (excluding dyspnoea emotions).

## 5. Conclusions

Fatigue is an important and highly prevalent extra-pulmonary symptom in patients with COPD. An inter-disciplinary PR programme reduces fatigue in COPD, and has a strong beneficial effect on fatigue (improvement ≥ MCID) in more than half the COPD patients who complete PR (even with severe or very severe COPD). Identification of prognostic factors for a clinically relevant improvement on fatigue following PR was not possible. Indeed, responders on fatigue had worse fatigue and HRQoL scores at baseline. Responders on fatigue were likely to be responders on other outcomes in PR. Future research should focus on identification of precipitating and perpetuating factors of fatigue and optimizing PR for those non-responders on fatigue with mild and severe fatigue at baseline.

## Figures and Tables

**Table 1:** Definitions and instruments of the sub-domains of the Nijmegen clinical screening instrument (NCSI).

Domain	Sub-Domain	Definition	Instruments/Measurement	Items
Symptoms	Subjective dyspnoea	The patient’s overall burden of pulmonary symptoms	PARS-D global dyspnea activity [31] PARS-D global dyspnea burden [31]	2
	Dyspnoea emotions	The level of frustration and anxiety a person experiences when dyspnoeic	DEQ frustration [31] DEQ anxiety [31]	6
	Fatigue	The level of experienced fatigue	CIS subjective fatigue [18]	8
Functional impairment	Subjective impairment	The experienced degree of impairment in general	QoLRiQ general activities [33,34]	4
	Behavioural impairment	The extent to which a person cannot perform specific and concrete activities as a result of having the disease	SIP home management [35] SIP ambulation [35]	22
Quality of life	General QoL	Mood and the satisfaction of a person with his/her life as a whole	BDI for primary care [36] Satisfaction with life scale [37]	12
	HRQoL	Satisfaction related to physical functioning and the future	Satisfaction physiological functioning [31] Satisfaction future [31]	2
	Satisfaction relations	Satisfaction with the (absent) relationships with spouse and others	Satisfaction spouse [31] Satisfaction social [31]	2

Abbreviations: PARS-D, physical activity rating scale-dyspnea; DEQ, dyspnea emotions questionnaire; CIS, checklist individual strength; QolRiQ, quality of life for respiratory illness questionnaire; QoL, quality of life; HRQoL, health-related quality of life; SIP, sickness impact profile; BDI, Beck depression inventory.

**Table 2:** Baseline characteristics of all eligible chronic obstructive pulmonary disease (COPD) patients referred for pulmonary rehabilitation (PR) (*n* = 446).

Demographical Features	
Age, y	60.5 ± 8.8
Male, *n* (%)	238 (53.4)
Education level, low/middle/high, *n* ^a^	229/151/60
Tobacco use, ^b^ non-/ex-/smoker, *n*	23/376/47
COPD diagnosis > 10 years, *n* (%) ^c^	145 (32.5)
≥1 self-reported comorbidity, *n* (%) ^b^	336 (76.2)
Clinical Features	
BMI, kg/m^2^	25.9 ± 5.5
BMI classification, Uw/No/Ow/Ob, *n*	69/157/140/80
FEV_1_, L	1.2 ± 0.5
FEV_1_, % predicted	42.5 ± 17.7
GOLD grade, I/II/III/IV, *n*	17/99/210/120
6MWD, m ^d^	383.2 ± 105.8
6MWD, % predicted ^d^	58.2 ± 15.4
<70% predicted, *n* (%)	290 (78.4)
Anxiety (SCL-90-A, 10–50), p ^b^	17.6 ± 7.2
Anxiety score ≥ 23, *n* (%)	95 (21.6)
Health Status (NCSI)	
Fatigue (CIS-Fatigue, 8–56), p	41.9 ± 9.3
Fatigue severity, normal/mild/severe, *n*	29/83/334
Dyspnoea (Dyspnoea VAS, 0–10), p	5.8 ± 1.9
HRQoL (2-10) *, p	5.8 ± 1.7
Depression (BDI-PC, 0–21) *, p	3.4 ± 3.0
Depression score ≥ 4, *n* (%)	172 (38.6)

Data is presented as mean ± SD, or number (%). * The Beck depression inventory for primary care (BDI-PC) is part of the domain ‘HRQoL’ (health-related quality of life). Alphabetic characters in superscript indicates a sample size deviant from *n* = 446, with ^a^ *n* = 440, ^b^ *n* = 441, ^c^ *n* = 403, ^d^ *n* = 370. Abbreviations: *n*, number; BMI, body mass index; Uw, underweight; No, normal weight; Ob, obese; Ow, overweight; FEV_1_, forced expiratory volume in first second; L, litre; GOLD, global initiative for chronic obstructive lung disease; 6MWD, six minute walking distance test; m, metre; SCL-90-A, 90-item symptom checklist—subscale anxiety; p, points; NCSI, Nijmegen clinical screening instrument; CIS-Fatigue, checklist individual strength—subscale subjective fatigue; Dyspnoea VAS, dyspnoea visual analogue scale; HRQoL, health-related quality of life; BDI-PC, Beck depression inventory for primary care.

**Table 3:** Baseline characteristics of COPD patients stratified for response of fatigue after PR (responder analysis, *n* = 417).

	Responders ^1^ (*n* = 233)	Non-Responders ^2^ (*n* = 184)	*p*-Value
Demographical Features			
Age, y	59.5 ± 8.8	61.8 ± 8.6	0.011
Male, *n* (%)	115 (49.4)	106 (57.6)	0.094
Tobacco use ^a^ non-/ex-/smoker, *n*	7/194/29	10/156/17	0.291
COPD diagnosis > 10 years, *n* (%) ^b^	82 (38.7)	54 (32.7)	0.233
≥1 self-reported comorbidity, *n* (%) ^a^	178 (77.4)	142 (77.6)	0.961
Clinical Features			
BMI, kg/m^2^	26.1 ± 5.6	25.9 ± 5.4	0.879
FFMi, kg/m^2 c^	16.4 ± 2.2	16.7 ± 2.4	0.220
FEV_1,_ L	1.3 ± 0.6	1.2 ± 0.5	0.217
FEV_1,_ % predicted	44.0 ± 18.0	41.4 ± 17.2	0.102
GOLD grade I/II/III/IV, *n*	10/58/106/59	6/36/91/51	0.543
6MWD, m ^d^	391.4 ± 105.0	369.0 ± 104.8	0.051
6MWD, % predicted ^d^	59.4 ± 14.9	56.4 ±16.1	0.072
<70 % predicted, *n* (%)	151 (76.7)	122 (81.9)	0.238
Quadriceps muscle strength, Nm ^e^	294.5 ± 102.9	284.9 ± 98.4	0.515
Anxiety (SCL-90-A, 10–50), *p* ^f^	17.7 ± 7.0	18.1 ± 7.5	0.620
Anxiety score ≥ 23, *n* (%)	48 (20.9)	44 (24.3)	0.406
NCSI—Symptoms			
Subjective dyspnoea, p ^#^	13.1 ± 3.8	13.3 ± 3.7	0.868
Dyspnoea (Dyspnoea VAS, 0–10) p ^#^	4.3 ± 1.9	5.4 ± 1.9	0.519
Dyspnoea emotions	13.0 ± 3.9	13.0 ± 4.1	0.926
Fatigue (CIS-Fatigue, 8–56), *p*	45.4 ± 7.3	40.5 ± 7.8	**<0.001**
Mild fatigue, *n* (%)	25 (10.7)	58 (31.5) ^ⱡ^	**<0.001**
Severe fatigue, *n* (%)	208 (89.3)	126 (68.5) ^ⱡ^	
NCSI—Quality of Life			
General QoL, p	28.0 ± 14.8	26.8 ± 14.3	0.615
HRQoL (2–10), p *	6.1 ± 1.6	5.7 ± 1.7	**0.004**
Depression (BDI-PC, 0–21), p *	3.6 ± 3.1	3.3 ± 2.9	0.384
Depression score ≥ 4, *n* (%)	93 (39.3)	74 (40.2)	0.950
Satisfaction with relations, p	4.0 ± 1.8	3.8 ±1.9	0.056
NCSI—Functional Impairment			
Subjective impairment, p	16.5 ± 5.2	16.9 ± 5.1	0.555
Behaviour impairment, p	27.1 ± 13.7	28.1 ± 14.3	0.517

Data is presented as mean ± SD, or number (%). *p*-value in bold indicates a significant difference. ^ⱡ^ indicates significant post-hoc pairwise comparison of the chi-square test (Bonferonni adjusted *p*-value < 0.01). * The BDI-PC is part of the subdomain ‘HRQoL’ of the NCSI. ^#^ The Dyspnoea VAS is part of the subdomain ‘subjective dyspnoea’ of the NCSI. ^1^ Δfatigue ≥ 10 points. ^2^ Δfatigue < 10 points. Alphabetic characters in superscript indicate a sample size deviant from *n* = 417, with ^a^ *n* = 413 (responders and non-responders respectively, 230 and 183), ^b^ *n* = 377 (resp., 212 and 165), ^c^ *n* = 396 (resp., 225 and 171), ^d^ *n* = 346 (resp., 197 and 149), ^e^ *n* = 371 (resp., 201 and 170), and ^f^ *n* = 411 (resp., 230 and 181). Abbreviations: COPD, chronic obstructive pulmonary disease; PR, pulmonary rehabilitation; *n*, number; BMI, body mass index; FFMi, fat-free mass index; FEV1, forced expiratory volume in first second; L, litre; GOLD, global initiative for chronic obstructive lung disease; 6MWD, six minute walking distance test; m, metre; Nm, Newton meter; SCL-90-A, 90-item symptom checklist—subscale anxiety; p, points; NCSI, Nijmegen clinical screening instrument; Dyspnoea VAS, dyspnoea visual analogue scale; CIS-Fatigue, checklist individual strength—subscale subjective fatigue; QoL, quality of life; HRQoL, health-related quality of life; BDI-PC, Beck depression inventory for primary care.

**Table 4:** Change scores after PR of patients with COPD stratified for response of fatigue.

	Responders ^1^ (*n* = 233)	Non-Responders ^2^ (*n* = 184)	*p*-Value
Clinical Features			
ΔBMI, kg/m^2 a^	−0.0 ± 1.4	−0.1 ± 1.3	0.914
ΔFFMi, kg/m^2 b^	0.3 ± 1.0	−0.1 ± 1.3	0.012
ΔFEV1, L ^c^	0.1 ± 0.3	0.0 ± 0.3	0.572
Δ6MWD, m ^d^	70.7 ± 74.4	38.3 ± 70.3	**0.001**
ΔQuadriceps muscle strength, Nm ^e^	25.5 ± 62.0	26.5 ± 68.7	0.901
ΔAnxiety (SCL-90-A, 10–50), p ^f^	−4.1 ± 5.5	−1.8 ± 6.0	**<0.001**
NCSI—Symptoms			
ΔSubjective dyspnoea, p ^# g^	−4.2 ± 4.2	−2.1 ± 4.3	**<0.001**
ΔDyspnoea (Dyspnoea VAS, 0–10), p ^# g^	−1.5 ± 2.1	−0.6 ± 2.0	**<0.001**
ΔDyspnoea emotions, p ^h^	−2.6 ± 3.9	−1.2 ± 3.7	**<0.001**
ΔFatigue (CIS-Fatigue, 8–56)	−19.2 ± 7.2	−1.7 ± 6.4	**<0.001**
NCSI—Quality of Life			
ΔGeneral QoL, p ^i^	−10.3 ± 12.3	−5.4 ± 12.0	**<0.001**
ΔHRQoL, p * ^i^	−2.5 ± 1.9	−1.1 ± 1.8	**<0.001**
ΔDepression (BDI-PC, 0–21), p *	−2.1 ± 2.6	−1.0 ± 2.6	**<0.001**
ΔSatisfaction with relationship, p	−0.9 ± 1.9	−0.2 ± 2.3	**0.003**
NCSI—Functional Impairment			
ΔSubjective impairment, p	−4.8 ± 5.6	−1.9 ± 5.0	**<0.001**
ΔBehaviour impairment, p	−2.9 ± 13.6	−0.6 ± 16.0	0.106

Data is presented as mean ± SD, or number (%). *p*-value in bold indicates a significant difference. * The BDI-PC is part of the subdomain ‘HRQoL’ of the NCSI. ^#^ The Dyspnoea VAS is part of the subdomain ‘subjective dyspnoea’ of the NCSI. ^1^ Δfatigue ≥ 10 points. ^2^ Δfatigue < 10 points. Alphabetic characters in superscript indicates a sample size deviant from *n* = 417, with ^a^ *n* = 363 (responders and non-responders respectively, 208 and 155), ^b^ *n* = 287 (resp., 167 and 120), ^c^ *n* = 347 (resp., 199 and 148), ^d^ *n* = 255 (resp. 155 and 100), ^e^ *n* = 291 (resp., 163 and 128), ^f^ *n* = 405 (resp., 227 and 178), ^g^ *n* = 416 (resp., 232 and 184), ^h^ *n* = 412 (resp., 229 and 183), and ^i^ *n* = 416 (resp., 233 and 183). Abbreviations: COPD, chronic obstructive pulmonary disease; PR, pulmonary rehabilitation; *n*, number; Δ, post-PR score minus pre-PR score; BMI, body mass index; FFMi, fat-free mass index; FEV1, forced expiratory volume in first second; L, litre; 6MWD, six minute walking distance test; m, metre; Nm, Newton meter; SCL-90-A, 90-item symptom checklist—subscale anxiety; p, points; NCSI, Nijmegen clinical screening instrument; Dyspnoea VAS, dyspnoea visual analogue scale; CIS-Fatigue, checklist individual strength—subscale subjective fatigue; QoL, quality of life; HRQoL, health-related quality of life; BDI-PC, Beck depression inventory for primary care.

## Supplementary Materials

The following are available online at https://www.mdpi.com/2077-0383/8/8/1264/s1, Table S1: Effectiveness of pulmonary rehabilitation at group level (*n* = 446), Table S2: Bivariate correlation between change in fatigue (Δfatigue) and change in other outcome measures.
