# Supplementary material for: Pulmonary Rehabilitation Reduces Subjective Fatigue in COPD: A Responder Analysis
Source: J Clin Med. 2019 Aug 20;8(8):1264. doi: 10.3390/jcm8081264 (PMC6722504; doi:10.3390/jcm8081264)
Supplement: Supplementary file 1 [file jcm-08-01264-s001.pdf]

Table S1. Effectiveness of pulmonary rehabilitation at group level (*n* = 446).

	**Pre-PR**	**Post-PR**	**Δ(post-pre)**	***p*-value**
Clinical features				
BMI, kg/m² [a]	25.9 ± 5.5	25.9 ± 5.0	-0.0 ± 1.3	0.744
FFMi, kg/m² [b]	16.5 ± 2.2	16.7 ± 2.1	0.1 ± 1.1	**<0.001**
FEV1, L [c]	1.2 ± 0.5	1.3 ± 0.7	0.1 ± 0.3	0.058
FEV1, % predicted [c]	42.6 ± 17.8	44.3 ± 20.0	1.7 ± 8.7	0.018
6MWD, m [d]	379.3 ± 102.6	436.9 ± 97.6	57.6 ± 73.2	**<0.001**
6MWD, % predicted d	58.0 ± 15.3	66.0 ± 14.9	7.9 ± 11.6	**<0.001**
Quadriceps muscle strength, Nm [e]	296.3 ± 105.2	322.3 ± 106.7	26.0 ± 64.0	**<0.001**
Anxiety (SCL-90-A, 10-50), p [f]	17.6 ± 7.2	14.7 ± 6.4	-3.0 ± 5.7	**<0.001**
NCSI – Symptoms				
Subjective dyspnoea, p [#] [g]	13.0 ± 3.8	9.8 ± 4.2	-3.2 ± 4.4	**<0.001**
Dyspnoea (Dyspnoea VAS, 0-10), p [#] [g]	5.8 ± 1.9	4.7 ± 2.0	-1.1 ± 2.1	**<0.001**
Dyspnoea emotions, p [h]	12.9 ± 4.0	11.0 ± 3.9	-1.9 ± 3.8	**<0.001**
Fatigue (CIS-Fatigue, 8-56), p	41.9 ± 9.3	31.5 ± 10.4	-10.4 ± 11.7	**<0.001**
NCSI – Quality of life				
General QoL, p [i]	26.8 ± 14.8	19.3 ± 12.3	-7.4 ± 12.5	**<0.001**
HRQoL (2-10), p * [i]	5.8 ±1 .7	4.0 ± 1.7	-1.8 ± 2.0	**<0.001**
Depression (BDI-PC, 0-21), p *	3.4 ± 3.0	1.9 ± 2.2	-1.5 ± 2.6	**<0.001**
Satisfaction with relations, p	3.8 ± 1.8	3.3 ± 1.6	-0.6 ± 2.1	**<0.001**
NCSI – Functional impairment				
Subjective impairment, p	16.4 ± 5.2	12.9 ± 5.1	-3.5 ± 5.5	**<0.001**
Behaviour impairment, p	27.2 ± 14.0	25.3 ± 14.5	-1.9 ± 14.6	**<0.001**

Data is presented as mean ±SD. P-value in bold indicates a significant difference. * The BDI-PC is part of the subdomain 'HRQoL' of the NCSI. [#] The Dyspnoea VAS is part of the subdomain 'Subjective dyspnoea' of the NCSI. Alphabetic characters in superscript indicates a sample size deviant from *n* = 446 with: [a] *n* = 389, [b] *n* = 310, [c] *n* = 373, [d] *n* = 270, [e] *n* = 311, [f] *n* = 434, [g] *n* = 445, [h] *n* = 440, and [i] *n* = 445. Abbreviations: PR, Pulmonary Rehabilitation; *n*, number; Δ, Post-PR score minus Pre-PR score; BMI, Body Mass Index; FFMi, Fat-Free Mass index; FEV1, Forced Expiratory Volume in first second; L, Litre; 6MWD, Six Minute Walking Distance test; m, metre; Nm, Newton meter; SCL-90-A, 90-items Symptom Checklist – Subscale Anxiety; p, points; NCSI, Nijmegen Clinical Screening Instrument; Dyspnoea VAS, Dyspnoea Visual Analogue Scale; CIS-Fatigue, Checklist Individual Strength – Subscale Subjective Fatigue; QoL, Quality of Life; HRQoL, Health-Related Quality of Life; BDI-PC, Beck Depression Inventory for Primary Care.

Table S2. Bivariate correlation between change in fatigue (Δfatigue) and change in other outcome measures.

	n	**Correlation coefficient**	***p*-Value**
Clinical features			
ΔBMI, kg/m^2	389	0.012	0.812
ΔFFMi, kg/m^2	310	-0.126	0.026
ΔFEV$_1$, % predicted	373	-0.053	0.303
Δ6MWD, % predicted	270	-0.323	**<0.001**
ΔLower limb strength, Nm	311	-0.035	0.537
ΔAnxiety (SCL-90-A), p	434	0.243	**<0.001**
NCSI—Symptoms			
ΔSubjective dyspnoea, p #	445	0.368	**<0.001**
ΔDyspnoea (Dyspnoea VAS), p #	445	0.321	**<0.001**
ΔDyspnoea-emotions, p	440	0.245	**<0.001**
NCSI—Quality of Life			
ΔGeneral QoL, p	445	0.300	**<0.001**
ΔHRQoL, p *	445	0.424	**<0.001**
ΔDepression (BDI-PC), p *	446	0.276	**<0.001**
ΔSatisfaction with relations, p	446	0.142	**0.003**
NCSI – Functional Impairment			
ΔBehaviour Impairment, p	446	0.131	**0.005**
ΔSubjective Impairment, p	446	0.306	**<0.001**

Data is presented as Pearson correlation coefficient unless otherwise stated. P-value in bold indicates a significant difference. * The BDI-PC is part of the subdomain 'HRQoL of the NCSI. # The Dyspnoea VAS is part of the subdomain 'Subjective dyspnoea' of the NCSI. Abbreviations: Δ, Post-PR score minus Pre-PR score; *n*, number; BMI, Body Mass Index; FFMi, Fat-Free Mass index; FEV1, Forced Expiratory Volume in first second; 6MWD, Six Minute Walking Distance test; Nm, Newton meter; SCL-90-A, 90items Symptom Checklist – Subscale Anxiety; p, points; NCSI, Nijmegen Clinical Screening Instrument; Dyspnoea VAS, Dyspnoea Visual Analogue Scale; QoL, Quality of Life; HRQoL, Health-Related Quality of Life; BDI-PC, Beck Depression Inventory for Primary Care.
